# Editorial: Musculoskeletal disorders related to dysparathyroidism

**DOI:** 10.3389/fnume.2026.1776939

**Published:** 2026-01-19

**Authors:** Emmanouil Panagiotidis, Jules Zhang-Yin

**Affiliations:** 1 Department of Nuclear Medicine, University of Thessaly, Volos, Greece; 2 Department of Nuclear Medicine, Cliniques du Sud Luxembourg, Arlon, Belgium

**Keywords:** CT, hyperparathyroidism, hypoparathyroidism, MRI, musculoskeletal, PET-CT, SPECT-CT, US

Editorial on the Research Topic Musculoskeletal disorders related to dysparathyroidism

Dysparathyroidism, whether due to excess or deficiency of parathyroid hormone (PTH), is a systemic endocrine disorder with a broad and often underestimated impact on the musculoskeletal system. Through its central role in calcium–phosphate balance and bone turnover, abnormal PTH activity affects skeletal integrity, muscle function, and periarticular tissues. While classical skeletal manifestations such as osteitis fibrosa cystica are well described, everyday clinical practice increasingly reveals more heterogeneous and subtle presentations. These are often overlooked or misinterpreted, especially in complex or comorbid patients. This Research Topic was therefore designed to bring together clinical, imaging, and therapeutic perspectives on musculoskeletal disorders related to dysparathyroidism, with emphasis on real-world diagnostic challenges.

One of the strongest messages emerging from this collection is the importance of modern imaging in understanding dysparathyroid bone disease. Conventional radiography and planar bone scintigraphy remain useful, but they often underestimate disease extent or fail to reflect underlying metabolic activity. Hybrid imaging techniques, such as PET/CT and SPECT/CT, allow whole-body evaluation of skeletal metabolism and provide functional information that complements morphology. The review and mini-review articles included in this Topic clearly show how tracers such as 18F-NaF, 18F-FDG, and 18F-fluorocholine can visualize both diffuse high-turnover bone disease and focal skeletal lesions, particularly in patients with long-standing or secondary hyperparathyroidism Panagiotidis et al., van der Bruggen and Bulten.

Diffuse skeletal uptake patterns, including the well-known “superscan”, reflect globally increased bone turnover driven by prolonged PTH excess. Recognizing these patterns is clinically relevant, as they offer insight into disease severity and systemic skeletal burden, often before irreversible structural changes become apparent. Importantly, functional imaging also allows assessment of skeletal response after treatment, whether medical, surgical, or interventional, and may support follow-up decisions in complex patients.

At the same time, dysparathyroidism may present with focal bone lesions, most notably brown tumors. These benign osteolytic lesions arise from excessive osteoclastic activity and reparative fibrosis but can closely resemble malignant bone disease on imaging. The case series included in this collection illustrates how 18F-fluorocholine PET/CT, interpreted together with biochemical findings, can accurately identify brown tumors and define their extent Zhang-Yin and Panagiotidis. This is particularly relevant in patients with multiple lesions or in those with a history of malignancy, where diagnostic uncertainty may lead to inappropriate staging or unnecessary treatment.

In this context, the inclusion of a large multicenter study on bone metastases in differentiated thyroid cancer adds an important clinical perspective. Although thyroid cancer is not a dysparathyroid disorder, bone metastases represent a critical differential diagnosis when evaluating skeletal lesions in patients with abnormal calcium metabolism or prior neck surgery. In daily practice, thyroid and parathyroid diseases often coexist. By describing clinical characteristics, prognostic factors, and survival outcomes, this study underlines the need for careful imaging interpretation and multidisciplinary discussion when distinguishing metabolic bone disease from true skeletal malignancy Dueñas-Disotuar et al.

Another recurring theme across the articles is the value of combining imaging modalities, rather than relying on a single technique. PET/CT offers high sensitivity and whole-body coverage, SPECT/CT provides accessible hybrid imaging with good skeletal detail, and conventional imaging still has a role in selected settings. Original research comparing conventional imaging with 18F-fluorocholine PET/CT for parathyroid adenoma localization supports this multimodality approach, showing how combined strategies can improve diagnostic confidence and assist in treatment planning in primary hyperparathyroidism Chiu et al.

Beyond imaging and diagnosis, this Research Topic also addresses therapeutic implications and their metabolic consequences. Interventions aimed at correcting hyperparathyroidism, whether surgical or minimally invasive, have direct effects on electrolyte balance and bone metabolism. Original research comparing radiofrequency ablation with parathyroidectomy in secondary hyperparathyroidism highlights that treatment choice may influence post-procedural biochemical complications. These findings reinforce the need for careful patient selection, close monitoring, and individualized management strategies. Treating dysparathyroidism is not only about normalizing PTH levels, but also about managing systemic musculoskeletal and metabolic effects.

Overall, the articles in this Research Topic show that musculoskeletal disorders related to dysparathyroidism represent a broad and interconnected spectrum. Diffuse bone remodeling, focal lesions, differential diagnostic challenges, and treatment-related changes are all influenced by disease duration, etiology, comorbidities, and therapeutic approach. Addressing this complexity requires close collaboration between endocrinologists, nuclear medicine physicians, radiologists, nephrologists, orthopedic surgeons, and oncologists.

In conclusion, this Research Topic brings together contemporary insights into the musculoskeletal impact of dysregulated PTH activity, with particular emphasis on imaging, differential diagnosis, and clinical relevance. By integrating reviews, original research, and illustrative case material, the collection aims to improve awareness of dysparathyroidism-related musculoskeletal disease and to support more informed, multidisciplinary patient care. We hope it will also stimulate further research in this evolving and clinically important field.

